# Supplementary material for: Prognostic Significance of Glucocorticoid Receptor Expression in Cancer: A Systematic Review and Meta-Analysis
Source: Cancers (Basel). 2021 Apr 1;13(7):1649. doi: 10.3390/cancers13071649 (PMC8037088; doi:10.3390/cancers13071649)
Supplement: Supplementary file 1 [file cancers-13-01649-s001.pdf]

cancers

Systematic Review

Prognostic Significance of Glucocorticoid Receptor Expression in Cancer: A Systematic Review and Meta-Analysis

Noor Bakour [1], Frank Moriarty [2], Gillian Moore [2], Tracy Robson [2] and Stephanie L. Annett [2,*]

[1] Department of Surgery, Trinity Translational Medicine Institute, Trinity College Dublin, Dublin 8, D08 W9RT, Ireland; bakourn@tcd.ie
[2] School of Pharmacy and Biomolecular Science, RCSI University of Medicine and Health Science, 123 St Stephen's Green, Dublin 2, D02 YN77, Ireland; frankmoriarty@rcsi.ie (F.M.); gillianmoore@rcsi.ie (G.M.); tracyrobson@rcsi.ie (T.R.)
* Correspondence: stephanieannett@rcsi.ie

Simple Summary: In solid tumours, emerging evidence indicates that signalling through the glucocorticoid receptor (GR) can encourage the growth and spread of tumours and so drugs targeting this receptor are in development for use in cancer treatment. For these reasons, GR may be useful in anticipating a patient's outcome upon their cancer diagnosis or to predict their tumours response to drugs targeting this receptor. In this review we aim to ascertain whether GR expression in tumours affects cancer patient survival. Overall, GR expression did not affect patient survival when assessing all cancer types. However, we found that in certain cancer subtypes such as gynaecological cancers (endometrial and ovarian) and early stage, untreated triple negative breast cancers, high GR expression is linked with cancer progression and therefore a poorer patient prognosis. Further studies are needed to uncover the exact role of GR in specific tumour (sub)types in order to provide the correct patients with GR targeting therapies.

Citation: Bakour, N.; Moriarty, F.; Moore, G.; Robson, T.; Annett, S.L. Prognostic Significance of Glucocorticoid Receptor Expression in Cancer: A Systematic Review and Meta-Analysis. *Cancers* **2021**, *13*, 1649. https://doi.org/10.3390/13071649

Academic Editor: Álvaro González

Received: 8 February 2021
Accepted: 24 March 2021
Published: 1 April 2021

Publisher's Note: MDPI stays neutral with regard to jurisdictional claims in published maps and institutional affiliations.

Copyright: © 2021 by the authors. Licensee MDPI, Basel, Switzerland. This article is an open access article distributed under the terms and conditions of the Creative Commons Attribution (CC BY) license (http://creativecommons.org/licenses/by/4.0/).

Table S1 Assessment of Bias Quality in Prognostic Studies (QUIPS) tool. Adapted from [1] and [2].

Bias domains	Items to be considered
1. Study population	- Were the selection criteria clearly defined?<br>- Similar point in the course of the disease?<br>- Was the study population representative of the population of interest?
Risk (low/moderate/high)	
2. Follow-up/attrition	- Was the follow-up period adequate?<br>- Was completeness of the follow-up described?<br>- Was completeness of the follow-up adequate?
Risk (low/moderate/high)	
3. Prognostic factor measurement	- Were prognostic factors clearly defined?<br>- Were prognostic factors measured appropriately?<br>- Were prognostic data available for an adequate proportion of the included participants?
Risk (low/moderate/high)	
4. Outcome measure	- Was the outcome of interest clearly defined?<br>- Was the outcome determined appropriately<br>- Were outcomes determined blind to prognostic information?
Risk (low/moderate/high)	
5. Analysis/confounding	- Were important confounding factors adequately accounted for?<br>- Were any treatments given to participants during the follow-up standardised or randomised?<br>- Were the analysis methods adequate?<br>- Was the reporting independent of results?<br>- Study free of other aspects that have potential risk of bias?
Risk (low/moderate/high)	

Table S2: Risk of bias assessment for included studies.

Study	Year	Study population	Follow up	PF measurement	Outcome measurement	Analysis/confounding
Abduljabbar et al. [3]	2015	low	low	low	moderate; aware of PF data	low
Allegra et al. [4]	1979	low	low	high; method unclear	moderate; not blinded	high; some adjuvant therapy
Chen et al. [5]	2015	moderate; no incl./excl., stages NR	low	low	moderate; aware of PF data	low
Elkashif et al. [6]	2020	low	low	high; no cut off	low	low
Gokon et al. [7]	2020	high; mixed stages and mets	moderate; follow up NR	low	low	moderate; surgery diff and other treatment unknown
Heuck et al. [8]	2012	low	low	low	moderate; aware of PF data	low
Ho et al. [9]	2002	high; stage	low	high; cut-off and indirect method	moderate; aware of PF data	low
Ip et al. [10]	2015	high; no incl./excl., stages	low	low	low	high; treatments differed
Ishiguro et al. [11]	2014	moderate; no incl./excl., stages, lymph node	moderate; follow up not reported	low	low	high; BCG not in analysis

Kanai et al. [12]	2020	low	low	low	moderate; aware of PF data	low
Kashiwagi et al. [13]	2016	high; no incl./excl., stages	moderate; not follow up	low	moderate; aware of PF data	moderate; treatment unknown
Kato, G. J et al. [14]	1993	low	low	high; no cut off and method	moderate; aware of PF data	low
Kost et al. [15]	2019	low	moderate; follow up unclear	low	low	moderate; treatment unknown
Lien et al. [16]	2008	high; incl/excl critera, varying stages, unsure if study pop represents actual pop	moderate; follow up not reported	low	moderate; outcome not clearly described, aware of PF data	high; confounding factors not reported/accounted for in analysis, non-sig data NR
Lu et al. [17]	2006	low	moderate; follow up not reported	low	moderate; aware of PF data	moderate; insignificant *p* values not reported
McNamara et al. [18]	2018	low	moderate; no follow up	low	moderate; aware of PF data	moderate; treatment unknown
Mimae et al. [19]	2011	moderate; incl./ecl. NR, stages	low	low	moderate; aware of PF data	moderate; treatment unknown
Mitani et al. [20]	2019	low	low	high; no cut off value	moderate; aware of PF data	moderate; treatment unknown
Pan et al. [21]	2011	moderate	moderate; follow up not reported	low	moderate; outcome unclear, PF data known	low
Puhr et al. [22]	2018	low	low	moderate; PF reported only in relapsed patients	moderate; aware of PF data	moderate; treatment unknown
Shi et al. [23]	2019	high; no patient info, different stages	moderate; follow up NR	moderate; measurement poorly described	moderate; aware of PF data	high; other confounding variables not reported/accounted for in analysis
Shim et al. [24]	2019	moderate; stages	moderate; follow up reporting unclear	low	moderate; aware of PF data	low
Surati et al. [25]	2011	high; stage and pop	moderate; follow up not reported	low	moderate; outcome not clearly described, aware of PF data	moderate; treatment unknown
Tangen et al. [26]	2017	low	low	low	low	high; adjuvant therapy not in analysis
Theocharis et al. [27]	2003	high; incl/excl critera, varying stages	low	low	low	low

Ueki et al. [28]	2020	high; incl./excl., stages	moderate; follow up NR	low	low	low
Vahrenkamp et al. [29]	2018	moderate; incl/excl NR, study pop characteristics NR	moderate; follow up not reported	moderate; PF not described in full, RNA seq used	moderate; aware of PF data	moderate; treatmnt unknown
Veneris et al. [30]	2017	low	low	low	moderate; aware of PF data	low
Veneris et al. [31]	2019	low	moderate; follow NR	low	moderate; aware of PF data	low
West et al. [32]	2016	high	low	low	moderate	low
West et al. [33]	2018	moderate; full patient data NR	moderate; follow up nr	low	moderate; aware of PF data	high; treatment not accounted for in analysis
Woenckhaus et al. [34]	2006	high; histology and stage	low	low	moderate; outcome not clearly described, aware of PF data	high; treatment not accounted for in analysis
Yakirevich et al.[35]	2011	low	low	low	high; survival analysis on CCRCC patients only, aware of PF data	low
Yeh et al. [36]	2006	low	low	high; cut off NR, GR method	moderate; aware of PF data	moderate
Zheng et al. [37]	2012	low	low	low	low	low

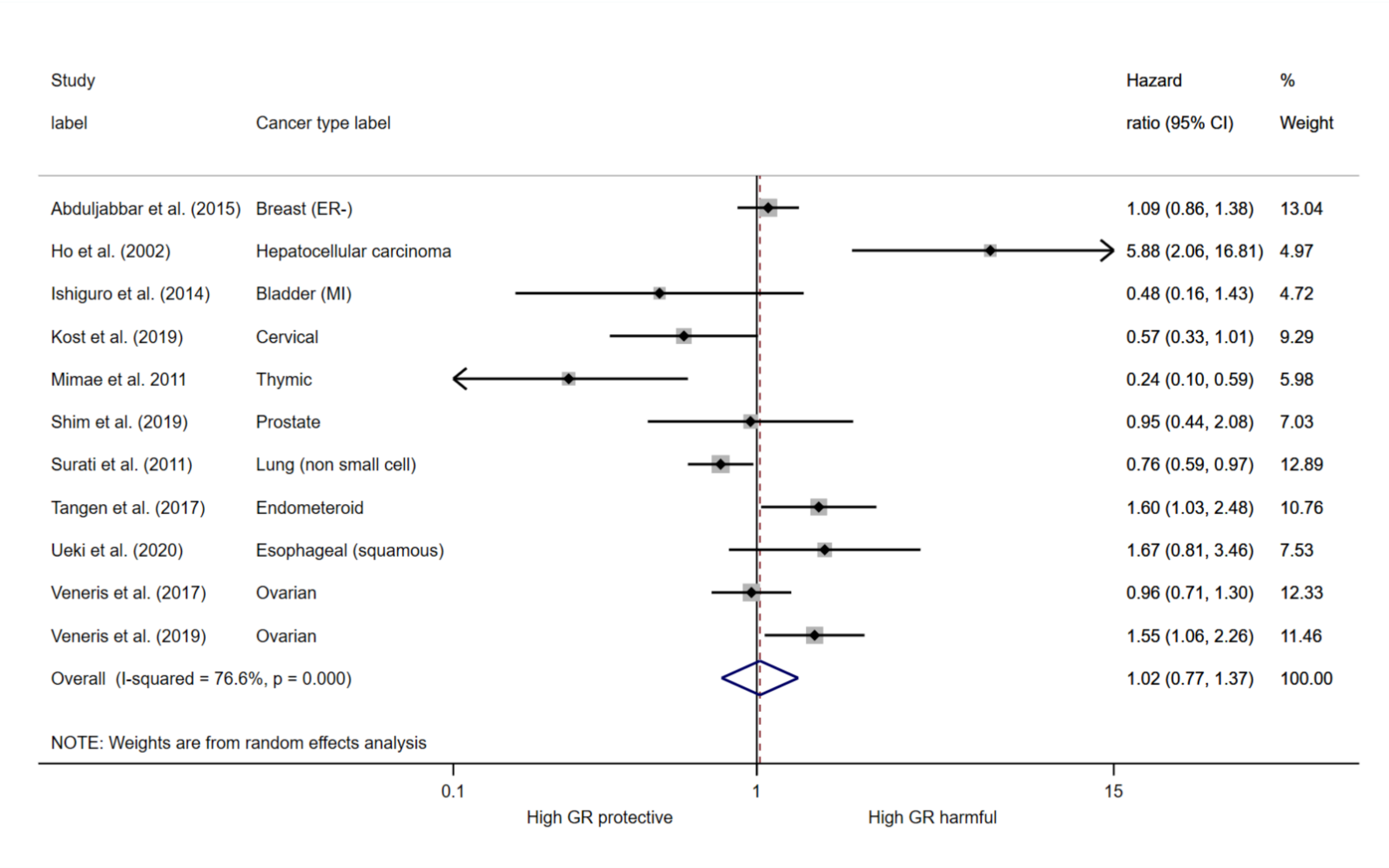

Figure S1 The forest plot for multivariate overall survival and glucocorticoid expression (*n*=2355)

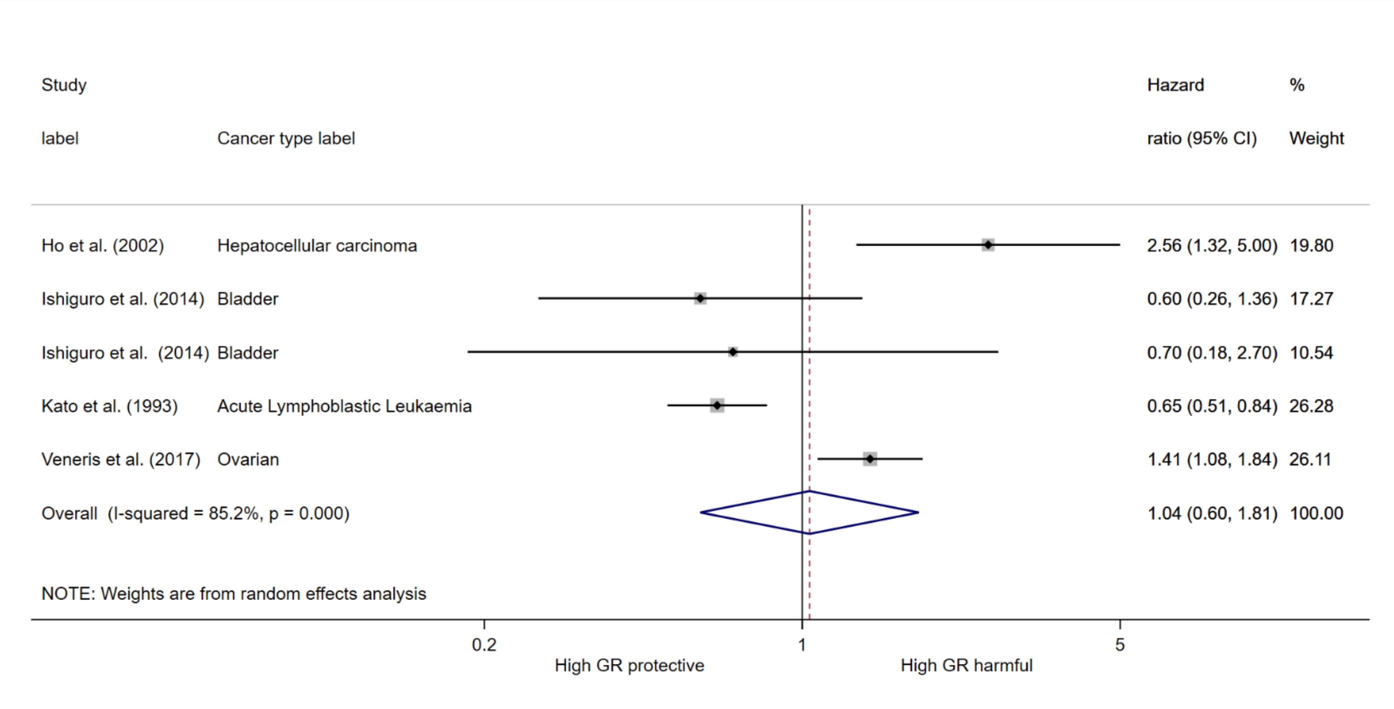

Figure S2 The forest plot for multivariate progression free survival and glucocorticoid expression (*n*=582)

A

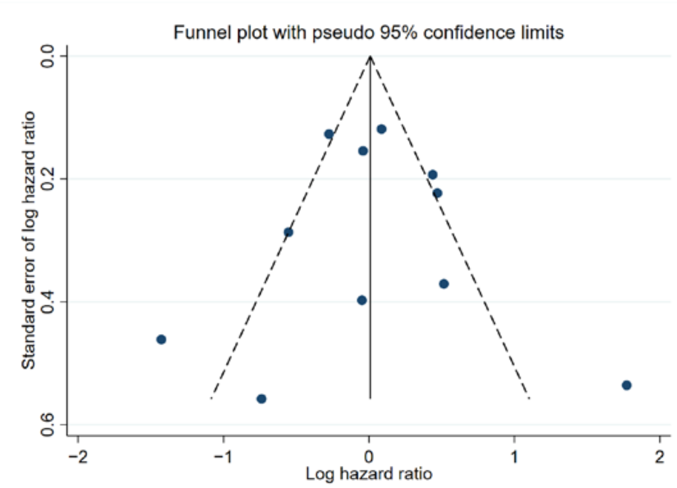

B

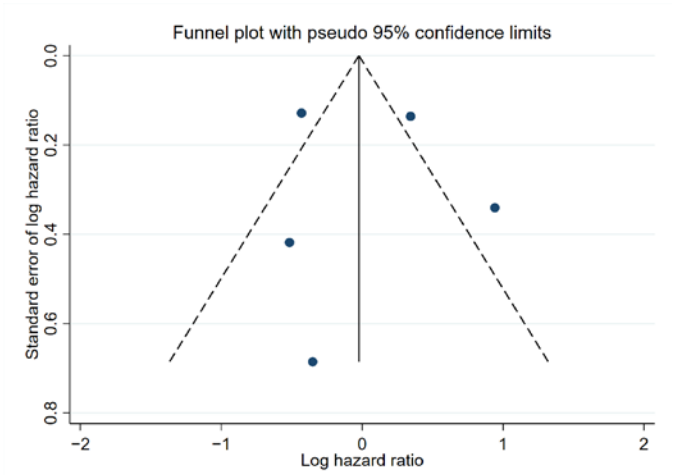

Figure S3 Funnel plot for **A**) multivariate overall survival and **B**) multivariate progression free survival meta analysis. The effect estimate (log hazard ratio) was plotted against the standard error of the effect estimate. Individual studies are represented by each point plotted on the graph. The black line centrally located on the graph represents the summary estimate of the effect of positive/high GR expression. The diagonal dotted lines represent the pseudo 95% confidence limits.

Table S2. Comparison of GR expression between normal and tumourous tissues. Seven studies assessed GR expression in normal tissues versus primary tumour or primary versus metastatic lesions or both. *Indicates studies where the exact proportion of samples expressing GR was not reported.

**Study**	**Cancer type**	**Normal vs Tumour**
Surati *et al.* [25]	Lung	Normal = Tumour*
Zheng *et al.* [37]	Bladder	GR high: 44% N and 21% T
Ishiguro *et al.* [11]	Bladder	GR+: 96% N and 87% T
Kashiwagi *et al.* [13]	Bladder	GR+: 84% N and 63% T
Puhr *et al.* [22]	Prostate	Normal > Tumour*
Kost *et al.* [15]	Cervical	Normal > Tumour*
**Study**	**Cancer type**	**Tumour vs Metastases**
Zheng *et al.* [37]	Bladder	GR high: 21% T and 0% M
Puhr *et al.* [22]	Prostate	Tumour < Metastases*
Tangen *et al.* [26]	Endometrial	GR+: 43% T and 78% M

Reference

1. Wolff, R.F.; Moons, K.G.M.; Riley, R.D.; Whiting, P.F.; Westwood, M.; Collins, G.S.; Reitsma, J.B.; Kleijnen, J.; Mallett, S. PROBAST: A Tool to Assess the Risk of Bias and Applicability of Prediction Model Studies. *Ann. Intern. Med.* **2019**, *170*, 51-58, doi:10.7326/m18-1376.
2. Hayden, J.A.; van der Windt, D.A.; Cartwright, J.L.; Côté, P.; Bombardier, C. Assessing bias in studies of prognostic factors. *Ann. Intern. Med.* **2013**, *158*, 280-286, doi:10.7326/0003-4819-158-4-201302190-00009.
3. Abduljabbar, R.; Negm, O.H.; Lai, C.F.; Jerjees, D.A.; Al-Kaabi, M.; Hamed, M.R.; Tighe, P.J.; Buluwela, L.; Mukherjee, A.; Green, A.R.; et al. Clinical and biological significance of glucocorticoid receptor (GR) expression in breast cancer. *Breast Cancer Res. Treat.* **2015**, *150*, 335-346, doi:10.1007/s10549-015-3335-1.
4. Allegra, J.C.; Lippman, M.E.; Simon, R.; Thompson, E.B.; Barlock, A.; Green, L.; Huff, K.K.; Do, H.M.; Aitken, S.C.; Warren, R. Association between steroid hormone receptor status and disease-free interval in breast cancer. *Cancer Treat. Rep.* **1979**, *63*, 1271-1277.
5. Chen, Z.; Lan, X.; Wu, D.; Sunkel, B.; Ye, Z.; Huang, J.; Liu, Z.; Clinton, S.K.; Jin, V.X.; Wang, Q. Ligand-dependent genomic function of glucocorticoid receptor in triple-negative breast cancer. *Nat. Commun.* **2015**, *6*, 8323, doi:10.1038/ncomms9323.
6. Elkashif, A.; Bingham, V.; Haddock, P.; Humphries, M.P.; McQuaid, S.; Mullan, P.B.; McCarthy, H.O.; Buckley, N.E. Glucocorticoid receptor expression predicts good outcome in response to taxane-free, anthracycline-based therapy in triple negative breast cancer. *J. Oncol.* **2020**, *2020*, 3712825, doi:10.1155/2020/3712825.
7. Gokon, Y.; Fujishima, F.; Taniyama, Y.; Ishida, H.; Yamagata, T.; Sawai, T.; Uzuki, M.; Ichikawa, H.; Itakura, Y.; Takahashi, K.; et al. Glucocorticoid receptor and serum- and glucocorticoid-induced kinase-1 in esophageal adenocarcinoma and adjacent Barrett's esophagus. *Pathol. Int.* **2020**, doi:10.1111/pin.12922.
8. Heuck, C.J.; Szymonifka, J.; Hansen, E.; Shaughnessy, J.D., Jr.; Usmani, S.Z.; van Rhee, F.; Anaissie, E.; Nair, B.; Waheed, S.; Alsayed, Y.; et al. Thalidomide in total therapy 2 overcomes inferior prognosis of myeloma with low expression of the glucocorticoid receptor gene NR3C1. *Clin. Cancer Res.* **2012**, *18*, 5499-5506, doi:10.1158/1078-0432.Ccr-12-0019.
9. Ho, W.L.; Wu, C.C.; Yeh, D.C.; Chen, J.T.; Huang, C.C.; Lin, Y.L.; Liu, T.J.; P'Eng F, K. Roles of the glucocorticoid receptor in resectable hepatocellular carcinoma. *Surgery* **2002**, *131*, 19-25, doi:10.1067/msy.2002.118710.
10. Ip, J.C.; Pang, T.; Glover, A.; Soon, P.; Zhao, J.; Clarke, S.; Robinson, B.G.; Gill, A.J.; Sidhu, S.B. Immunohistochemical validation of overexpressed genes identified by global expression microarrays in adrenocortical carcinoma reveals potential predictive and prognostic biomarkers. *Oncologist* **2015**, *20*, 247-256, doi:10.1634/theoncologist.2014-0392.
11. Ishiguro, H.; Kawahara, T.; Zheng, Y.; Netto, G.J.; Miyamoto, H. Reduced glucocorticoid receptor expression predicts bladder tumor recurrence and progression. *Am. J. Clin. Pathol.* **2014**, *142*, 157-164, doi:10.1309/ajcpu8ucezyg4wtv.
12. Kanai, A.; McNamara, K.M.; Iwabuchi, E.; Miki, Y.; Onodera, Y.; Guestini, F.; Khalid, F.; Sagara, Y.; Ohi, Y.; Rai, Y.; et al. Significance of glucocorticoid signaling in triple-negative breast cancer patients: a newly revealed interaction with androgen signaling. *Breast Cancer Res. Treat.* **2020**, *180*, 97-110, doi:10.1007/s10549-020-05523-7.
13. Kashiwagi, E.; Fujita, K.; Yamaguchi, S.; Fushimi, H.; Ide, H.; Inoue, S.; Mizushima, T.; Reis, L.O.; Sharma, R.; Netto, G.J.; et al. Expression of steroid hormone receptors and its prognostic significance in urothelial carcinoma of the upper urinary tract. *Cancer Biol. Ther.* **2016**, *17*, 1188-1196, doi:10.1080/15384047.2016.1235667.
14. Kato, G.J.; Quddus, F.F.; Shuster, J.J.; Boyett, J.; Pullen, J.D.; Borowitz, M.J.; Whitehead, V.M.; Crist, W.M.; Leventhal, B.G. High glucocorticoid receptor content of leukemic blasts is a favorable prognostic factor in childhood acute lymphoblastic leukemia. *Blood* **1993**, *82*, 2304-2309.
15. Kost, B.P.; Beyer, S.; Schroder, L.; Zhou, J.; Mayr, D.; Kuhn, C.; Schulze, S.; Hofmann, S.; Mahner, S.; Jeschke, U.; et al. Glucocorticoid receptor in cervical cancer: an immunhistochemical analysis. *Arch. Gynecol. Obstet.* **2019**, *299*, 203-209, doi:10.1007/s00404-018-4928-9.
16. Lien, H.C.; Lu, Y.S.; Shun, C.T.; Yao, Y.T.; Chang, W.C.; Cheng, A.L. Differential expression of glucocorticoid receptor in carcinomas of the human digestive system. *Histopathology* **2008**, *52*, 314-324, doi:10.1111/j.1365-2559.2007.02953.x.
17. Lu, Y.S.; Lien, H.C.; Yeh, P.Y.; Kuo, S.H.; Chang, W.C.; Kuo, M.L.; Cheng, A.L. Glucocorticoid receptor expression in advanced non-small cell lung cancer: clinicopathological correlation and in vitro effect of glucocorticoid on cell growth and chemosensitivity. *Lung Cancer* **2006**, *53*, 303-310, doi:10.1016/j.lungcan.2006.05.005.
18. McNamara, K.M.; Guestini, F.; Sauer, T.; Touma, J.; Bukholm, I.R.; Lindstrom, J.C.; Sasano, H.; Geisler, J. In breast cancer subtypes steroid sulfatase (STS) is associated with less aggressive tumour characteristics. *Br. J. Cancer* **2018**, *118*, 1208-1216, doi:10.1038/s41416-018-0034-9.
19. Mimae, T.; Tsuta, K.; Takahashi, F.; Yoshida, A.; Kondo, T.; Murakami, Y.; Okada, M.; Takeuchi, M.; Asamura, H.; Tsuda, H. Steroid receptor expression in thymomas and thymic carcinomas. *Cancer* **2011**, *117*, 4396-4405, doi:10.1002/cncr.26061.
20. Mitani, Y.; Lin, S.H.; Pytynia, K.B.; Ferrarotto, R.; El-Naggar, A.K. Reciprocal and autonomous glucocorticoid and androgen receptor activation in salivary duct carcinoma. *Clin. Cancer Res.* **2020**, *26*, 1175-1184, doi:10.1158/1078-0432.Ccr-19-1603.
21. Pan, D.; Kocherginsky, M.; Conzen, S.D. Activation of the glucocorticoid receptor is associated with poor prognosis in estrogen receptor-negative breast cancer. *Cancer Res.* **2011**, *71*, 6360-6370, doi:10.1158/0008-5472.Can-11-0362.
22. Puhr, M.; Hoefer, J.; Eigentler, A.; Ploner, C.; Handle, F.; Schaefer, G.; Kroon, J.; Leo, A.; Heidegger, I.; Eder, I.; et al. The glucocorticoid receptor is a key player for prostate cancer cell survival and a target for improved antiandrogen therapy. *Clin. Cancer Res.* **2018**, *24*, 927-938, doi:10.1158/1078-0432.ccr-17-0989.

23. Shi, W.; Wang, D.; Yuan, X.; Liu, Y.; Guo, X.; Li, J.; Song, J. Glucocorticoid receptor-IRS-1 axis controls EMT and the metastasis of breast cancers. *J. Mol. Cell Biol.* **2019**, *11*, 1042-1055, doi:10.1093/jmcb/mjz001.
24. Shim, M.; Kim, Y.; Park, Y.; Ahn, H. Taxane-based chemotherapy induced androgen receptor splice variant 7 in patients with castration-resistant prostate cancer: a tissue-based analysis. *Sci. Rep.* **2019**, *9*, 16794, doi:10.1038/s41598-019-53280-5.
25. Surati, M.; Robinson, M.; Nandi, S.; Faoro, L.; Demchuk, C.; Rolle, C.E.; Kanteti, R.; Ferguson, B.D.; Hasina, R.; Gangadhar, T.C.; et al. Proteomic characterization of non-small cell lung cancer in a comprehensive translational thoracic oncology database. *J. Clin. Bioinforma.* **2011**, *1*, 1-11, doi:10.1186/2043-9113-1-8.
26. Tangen, I.L.; Veneris, J.T.; Halle, M.K.; Werner, H.M.; Trovik, J.; Akslen, L.A.; Salvesen, H.B.; Conzen, S.D.; Fleming, G.F.; Krakstad, C. Expression of glucocorticoid receptor is associated with aggressive primary endometrial cancer and increases from primary to metastatic lesions. *Gynecol. Oncol.* **2017**, *147*, 672-677, doi:10.1016/j.ygyno.2017.09.013.
27. Theocharis, S.; Kouraklis, G.; Margeli, A.; Agapitos, E.; Ninos, S.; Karatzas, G.; Koutselinis, A. Glucocorticoid receptor (GR) immunohistochemical expression is correlated with cell cycle-related molecules in human colon cancer. *Dig. Dis. Sci.* **2003**, *48*, 1745-1750, doi:10.1023/a:1025578527978.
28. Ueki, S.; Fujishima, F.; Kumagai, T.; Ishida, H.; Okamoto, H.; Takaya, K.; Sato, C.; Taniyma, Y.; Kamei, T.; Sasano, H. GR, Sgk1, and NDRG1 in esophageal squamous cell carcinoma: their correlation with therapeutic outcome of neoadjuvant chemotherapy. *BMC Cancer* **2020**, *20*, 161, doi:10.1186/s12885-020-6652-7.
29. Vahrenkamp, J.M.; Yang, C.H.; Rodriguez, A.C.; Almomen, A.; Berrett, K.C.; Trujillo, A.N.; Guillen, K.P.; Welm, B.E.; Jarboe, E.A.; Janat-Amsbury, M.M.; et al. Clinical and genomic crosstalk between glucocorticoid receptor and estrogen receptor alpha in endometrial cancer. *Cell Rep.* **2018**, *22*, 2995-3005, doi:10.1016/j.celrep.2018.02.076.
30. Veneris, J.T.; Darcy, K.M.; Mhawech-Fauceglia, P.; Tian, C.; Lengyel, E.; Lastra, R.R.; Pejovic, T.; Conzen, S.D.; Fleming, G.F. High glucocorticoid receptor expression predicts short progression-free survival in ovarian cancer. *Gynecol. Oncol.* **2017**, *146*, 153-160, doi:10.1016/j.ygyno.2017.04.012.
31. Veneris, J.T.; Huang, L.; Churpek, J.E.; Conzen, S.D.; Fleming, G.F. Glucocorticoid receptor expression is associated with inferior overall survival independent of BRCA mutation status in ovarian cancer. *Int. J. Gynecol. Cancer* **2019**, doi:10.1136/ijgc-2018-000101.
32. West, D.C.; Pan, D.; Tonsing-Carter, E.Y.; Hernandez, K.M.; Pierce, C.F.; Styke, S.C.; Bowie, K.R.; Garcia, T.I.; Kocherginsky, M.; Conzen, S.D. GR and ER coactivation alters the expression of differentiation genes and associates with improved ER+ breast cancer outcome. *Mol. Cancer Res.* **2016**, *14*, 707-719, doi:10.1158/1541-7786.Mcr-15-0433.
33. West, D.C.; Kocherginsky, M.; Tonsing-Carter, E.Y.; Dolcen, D.N.; Hosfield, D.J.; Lastra, R.R.; Sinnwell, J.P.; Thompson, K.J.; Bowie, K.R.; Harkless, R.V.; et al. Discovery of a glucocorticoid receptor (GR) activity signature using selective GR antagonism in ER-negative breast cancer. *Clin. Cancer Res.* **2018**, *24*, 3433-3446, doi:10.1158/1078-0432.ccr-17-2793.
34. Woenckhaus, J.; Franke, F.E.; Hackethal, A.; Von Georgi, R.; Münstedt, K. Glucocorticosteroid receptors in ovarian carcinomas. *Oncol. Rep.* **2006**, *15*, 1137-1140.
35. Yakirevich, E.; Matoso, A.; Sabo, E.; Wang, L.J.; Tavares, R.; Meitner, P.; Morris, D.J.; Pareek, G.; Delellis, R.A.; Resnick, M.B. Expression of the glucocorticoid receptor in renal cell neoplasms: an immunohistochemical and quantitative reverse transcriptase polymerase chain reaction study. *Hum. Pathol.* **2011**, *42*, 1684-1692, doi:10.1016/j.humpath.2011.01.014.
36. Yeh, D.C.; Cheng, S.B.; Yu, C.C.; Ho, W.L.; Wu, C.C.; Liu, T.J.; P'Eng F, K. Role of glucocorticoid receptor in serosa-involved gastric carcinoma after gastrectomy. *J. Gastrointest. Surg.* **2006**, *10*, 706-711, doi:10.1016/j.gassur.2005.10.004.
37. Zheng, Y.; Izumi, K.; Li, Y.; Ishiguro, H.; Miyamoto, H. Contrary regulation of bladder cancer cell proliferation and invasion by dexamethasone-mediated glucocorticoid receptor signals. *Mol. Cancer Ther.* **2012**, *11*, 2621-2632, doi:10.1158/1535-7163.Mct-12-0621.
